# Full-length genome sequence analysis of enzootic nasal tumor virus isolated from goats in China

**DOI:** 10.1186/s12985-017-0795-4

**Published:** 2017-07-26

**Authors:** Yapeng He, Qi Zhang, Jing Wang, Man Zhou, Mingzhe Fu, Xingang Xu

**Affiliations:** College of Veterinary Medicine, Northwest A&F University, Yangling, Shaanxi 712100 People’s Republic of China

**Keywords:** Enzootic nasal tumor virus, Betaretrovirus, Pathology, Genome sequence, Phylogenetic analysis

## Abstract

**Background:**

Enzootic nasal tumor virus (ENTV) is a betaretrovirus of sheep (ENTV-1) and goats (ENTV-2) associated with neoplastic transformation of epithelial cells of the ethmoid turbinate. Confirmation of the role of ENTV in the pathogenesis of enzootic nasal adenocarcinoma (ENA) has yet to be resolved due to the inability to culture the virus. Very little is known about the prevalence of this disease, particularly in China.

**Methods:**

To evaluate the genetic diversity of ENTV-2 from Shaanxi province of China, the complete genome sequence of four isolates from Shaanxi province was determined by RT-PCR. These sequences were analyzed to evaluate their genetic relatedness with other small ruminant betaretroviruses. Phylogenetic analyses based on the gag gene and env gene were performed.

**Results:**

The ENTV-2-Shaanxi1 genome shared 97.0% sequence identity with ENTV-2-SC (accession number HM104174.1), and 89.6% sequence identity with the ENTV-2 sequences (accession number AY197548.1). ENTV-2 is closely related to the ENTV-1 and jaagsiekte retrovirus (JSRV). The main sequence differences between these viruses reside in LTR, two small regions of Gag, Orf-x, and the transmembrane (TM) region of Env. A stretch of 6 consecutive proline residues exists in VR1 of the ENTV-2-Shaanxi1 ~ 4 isolates. All the ENTV-2-Shaanxi isolates have the YXXM motif in the cytoplasmic tail of the Env. Phylogenetic analysis by nucleotide sequences showed that ENTV-2-Shaanxi1 ~ 4 isolates were closest related to two ENTV-2 isolates published in NCBI, especially with ENTV-2-SC strain.

**Conclusions:**

This finding indicates that ENA most likely was introduced to Shaanxi province by the movement of contaminated goats from other areas in China. This study adds to understand the circulation, variation and distribution of ENTV-2, and may prove beneficial in future control or eradication programmes.

## Background

Enzootic nasal adenocarcinoma (ENA) of sheep and goats and ovine pulmonary adenocarcinoma (OPA or jaagsiekte) are contagious diseases characterized by neoplastic transformation of secretory epithelial cells in the respiratory tract [1, 2]. OPA is a tumour derived from type II pneumocytes and Clara cells in the lung, whereas ENA arises from secretory epithelial cells of the ethmoid turbinate [3]. The gross pathology and histology of ENA in sheep and goats appear to be identical [1, 4, 5]. Jaagsiekte sheep retrovirus (JSRV), an ovine betaretrovirus, is the causative agent of OPA [2]. Enzootic nasal adenocarcinoma (ENA) is an economically important contagious tumour of the nasal mucosa of sheep and goats and is associated with enzootic nasal tumour virus (ENTV) [3–5]. ENTV belongs to the genus *Betaretrovirus* in the family *Retroviridae*. ENTV display characteristics of both D- and B-type viruses. The ENTV genome consists of a single, positive stranded RNA of about 7.5 kb and has a structure analogous to that of cellular mRNA, with 5′ and 3′ untranslated regions (UTR), a 7-methylguanosine cap and a polyadenylated 3′ end. Like other retroviral genomes, ENTV genome has a basic canonical structure, 5′-U5-Gag-Pro-Pol-Env-U3-3′, with four open reading frames (Orf) and flanking untranslated regions and terminal repeats (LTR) on either end. The *gag* (group antigen) gene encodes the structural proteins that make up the capsid (CA) and matrix (MA) layer or shell, as well as the nucleocapsid (NC) protein, which interacts directly with the genomic RNA. The *pro* gene encodes the viral protease (PR) responsible for proteolytic processing of viral proteins. The *pol* gene encodes reverse transcriptase (RT) and integrase (IN), the replicative enzymes required for reverse transcription and integration of the genome. The *env* gene encodes both SU (surface) and TM (transmembrane) subunits of the envelope glycoprotein [6]. With the exception of Australia and New Zealand, ENA has been recorded worldwide wherever sheep and goats are farmed, with a prevalence of up to 10% in some areas [4, 6]. Very little is known about the prevalence of this disease due to the lack of an infectious molecular clone and the inability to culture the virus, and only two full-length sequences are available for ENTV-2 (ENTV-2 European strain: accession number AY197548.1, and Chinese ENTV-2-SC strain: accession number HM104174.1) [1, 7, 8].

Goat enzootic nasal tumors appeared enzootically in China in recent years. Clinically, the affected goats showed copious serous nasal discharge, then snuffle and progressively developed dyspnea, ocular protrusion and skull deformations, eventually death from suffocation. The epidemiology, clinical and pathohistological pattern of goat intranasal adenoma and adenocarcinoma in Shaanxi province were similar to those of goat enzootic intranasal tumors that had in Spain, France and other provinces of China [1]. In order to understand the molecular evolution of ENTV-2, the full length genome sequence of four ENTV-2 derived from nasal fluid of ENA isolated from conventionally reared goats in China was determined and analyzed with other retroviruses. The results showed that the main sequence differences between these viruses reside in LTR, two small regions of Gag, Orf-x, and the transmembrane (TM) region of Env. Phylogenetic analysis revealed that these Shaanxi isolates were closely related to all the known ENTV-2 isolates, especially with ENTV-2-SC strain.

## Methods

### Clinical samples

Four goats exhibiting clinical signs of ENA were obtained from four geographically distinct flocks in Shaanxi province, China (Table 1). Nasal fluid, serum and tissue samples (nasal tumor and adjacent unaffected nasal turbinate, trachea, lung, heart, spleen, liver and kidney) were collected and flash frozen in liquid nitrogen or preserved in freezer at −80 °C. Nasal fluids from disease-free goats were used as negative controls.

**Table 1:** The information of the 4 ENTV-2

Goat number	Isolate name	Etiological agent	Date, origin	Accession number
1	ENTV-2-Shaanxi1	ENTV-2	2015, Yangling, Shaanxi	KU179192
2	ENTV-2-Shaanxi2	ENTV-2	2016, Baoji, Shaanxi	KU980910
3	ENTV-2-Shaanxi3	ENTV-2	2016, Yangling, Shaanxi	KU980911
4	ENTV-2-Shaanxi4	ENTV-2	2016, Lantian, Shaanxi	KU980912

### RNA extraction and RT-PCR

ENTV-2 ware purified from nasal fluids by isopycnic centrifugation as described previously [9–11].

Viral RNA were extracted from the purified virus using the TIANamp Virus RNA Kit (Tiangen, Beijing, China) according to the manufacture’s protocol, and cDNA was synthesized by oligo(dT)-primed or random-primed reverse transcription, using the Moloney murine leukaemia virus M-MLV (H-) riboclone cDNA synthesis system (Promega, Madison, USA). The cDNA was used as a template for subsequent PCR analysis.

Five pairs of primers were designed and synthesized according to ENTV-2 sequences (accession number AY197548.1) (Table 2) [1, 8]. PCRs were performed with a final reaction volume of 50 μL, containing 25 μL 2 × Pfu MasterMix (Providing DNA Polymerase, PCR Buffer, Mg2+, dNTPs; Cwbio, Beijing, China), 5 μL cDNA, 1 μL each of the primers (20 pmol) and RNase-free water. Cycling conditions were as follows: 94 °C for 3 min followed by 30 cycles of 94 °C for 30 s, 55 °C for 30 s and 72 °C for 70 s. A final extension of 5 min at 72 °C concluded the program. PCR products were purified with EasyPure Quick Gel Extraction Kit (Transgen, Beijing, China). Purified PCR products were cloned into the pEASY-T1 Cloning vector (Transgen, Beijing, China) following the manufacturers instructions and purified plasmid DNA and sequenced at BGI (Beijing, China).

**Table 2:** Primers for amplifying the complete genome of ENTV-2

Primer	Primer sequence(5′-3′)	Location	Product length
1F	ACAAGGCATCAGCCATTTTGGTCTGATCCTCTCAACCCCA	1-40	627 bp
1R	AGGAGGAGGAGCATCATAACCAGGCTCTGGGTCAGGAATA	627-588	
2F	GTTTTCCTCGCCACTACTCTTG	151-172	2249 bp
2R	TACCCAATAAGCGTCGGATGAT	2399-2378	
3F	CACTCCTAATTTGTGCCCACG	1848-1869	1087 bp
3R	GGCCACTGATCGACCCATAC	2934-2915	
4F	GAAGAGGTTTGGGGTGTTTTCCCTAGGGACCTCTGATTCTCCTGTGAC	2830-2878	2258 bp
4R	GTTTAAGACGTTGATGAGCTCGTTCTACAATCCCTTGTCCCTGTGGGT	5087-5040	
5F	AGAACGAGCTCATCAACGTCTTAAACATCAACT	5062-5094	2379 bp
5R	CTTGTTGTTTTATTGTGTCATAGTATATAT	7440-7411	

### Sequence and phylogenetic analysis

Full-length genomes were assembled using Lasergene v7.1 sequence analysis software package (DNASTAR Inc. Madison, WI). Nucleotide sequence editing, analysis, prediction of amino acid sequences and alignments were conducted using the DNASTAR software (DNASTAR Inc. Madison, WI). Pairwise sequence alignments were carried out by using Jotun Hein method. The unrooted phylogenetic trees were generated by the distance-based neighbor-joining method using MegAlign program in DNASTAR (DNASTAR Inc. Madison, WI) by comparison of the nucleotide sequences of *gag* and *env* gene. In addition to four isolates in this study, 20 previously reported retroviruses reference strains sequences were also included for comparison (Table 3).

**Table 3:** Profile of betaretroviruses isolates used for sequence analyses

Etiological agent	Isolate name	Date, origin	Host	Accession number
ENTV-2	ENTV-2	2003, Spain	Goat	AY197548.1
ENTV-2	ENTV-2-SC	2008, China	Goat	HM104174.1
ENTV-1	ENTV-1	1999, UK	Sheep	NC007015.1
ENTV-1	ENTV-1-1NA1	2009, Canada	Sheep	GU292317.1
ENTV-1	ENTV-1-1NA2	2008, Canada	Sheep	GU292315.1
ENTV-1	ENTV-1-1NA3	2008, Canada	Sheep	GU292318.1
ENTV-1	ENTV-1-1NA4	2008, Canada	Sheep	FJ744146.1
ENTV-1	ENTV-1-1NA5	2008, Canada	Sheep	GU292316.1
ENTV-1	ENTV-1-1NA6	2008, Canada	Sheep	FJ744149.1
ENTV-1	ENTV-1-1NA7	2008, Canada	Sheep	FJ744150.1
ENTV-1	ENTV-1-1NA8	2008, Canada	Sheep	FJ744147.1
ENTV-1	ENTV-1-1NA9	2008, Canada	Sheep	FJ744148.1
ENTV-1	ENTV-1-1NA10	2009, USA	Sheep	GU292314.1
JSRV	JSRV-C1/China	2013, China	Sheep	KP691837.1
JSRV	JSRV-Inner Mongolia	2013, China	Sheep	DQ838494.1
JSRV	JSRV-USA	2009, USA	Sheep	NC-001494.1
JSRV	JSRV-NM	2012, China	Sheep	JQ837489.1
JSRV	JSRV-SD	2014, China	Sheep	KC691273.1

### Nucleotide sequence accession numbers

The information of goats with nasal tumors was in Table 1. Goat 1 and 3 were collected at different time points in the same farm in Yangling city, thereby making it possible to assess the level of ENTV-2 nucleotide divergence within a given farm. All of the sequences described in this manuscript are available in the GenBank Nucleotide Sequence Database under the accession numbers found in Table 1.

## Results

### Clinical findings and gross pathology

**Fig. 1:**
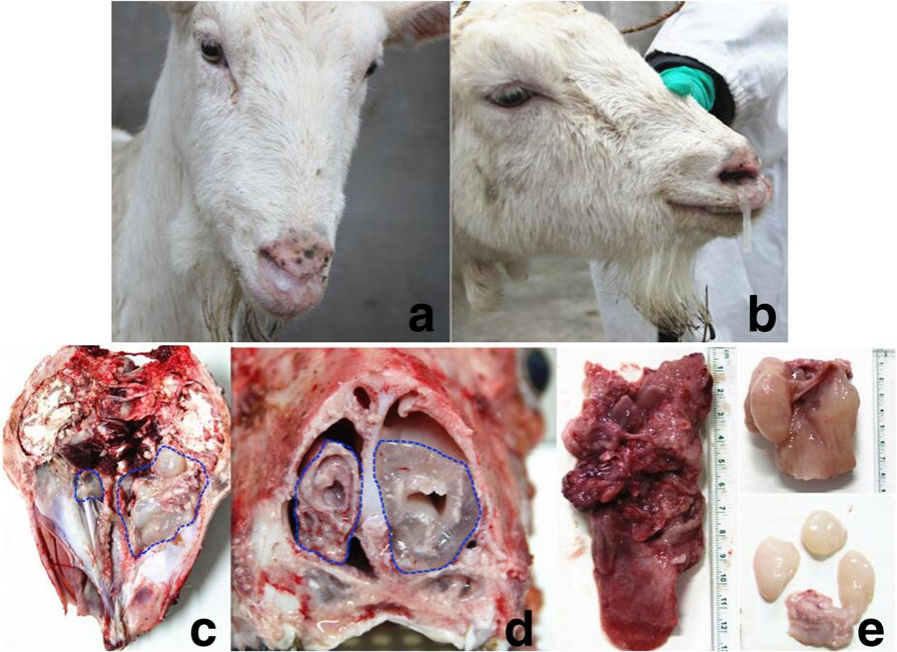
Gross photograph of ENA affected goats. **a** and **b**, Viscous purulent nasal fluid of goat infected with ENTV. Macroscopic findings in goat naturally infected with ENTV. Tumor masses are demarcated by dotted lines; **c**, Unilateral mass in the cranial nasal cavity with a soft, gelatinous consistency; **d**, Sagittal section of the nasal cavity of a goat with ENA. Bilateral tumor in the nasal cavity; **e**, The size of the tumors removed from the nasal cavity of the goats with ENA

The goats infected with ENTV showed weight loss, chronic nasal discharge, difficulty breathing and viscous purulent nasal fluid (Fig. 1a & b). In all cases, the goats were examined at necropsy and confirmed to have nasal adenocarcinoma (Fig. 1c & d). In general, the cranial part of the mass appeared white and gelatinous, while the caudal part of the mass tended to be more nodular with brownish-red areas of hemorrhage (Fig. 1c). The typical postmortem finding was a mass in the ethmoidal area of the nasal cavity, usually bilateral, ranging from 1 to 15 cm length (Fig. 1e). Even a few small adenomas can fall off from the nasal cavity (Fig. 1e). No lesions were observed in lung and any other organ with the exception of the trachea, which was occasionally filled with white froth.

### Sequencing ENTV-2

**Fig. 2:**
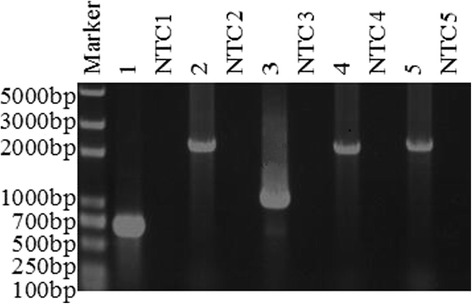
A representative gel of the resulting PCR products. The cDNA made from ENTV RNA was used as a template in the 1, 2. 3, 4, and 5 lane, and cDNA made from healthy goats nasal fluids were used as a template in the NTC1 ~ 5 lane. 1 ~ 5 applied the primers1, 2, 3, 4, and 5, respectively. NTC, negative control

The complete viral genomes of ENTV-2 were obtained from nasal fluids using 5 pairs of primers designed to selectively amplify exogenous ENTV-2 (Table 2; Fig. 2). RNA isolated from nasal fluids of healthy goat was used as a negative control (Fig. 2). A consensus sequence for the China ENTV-2 genomes were determined and named ENTV-2-Shaanxi1, ENTV-2-Shaanxi2, ENTV-2-Shaanxi3, ENTV-2-Shaanxi4. The nucleotide sequences from 4 different isolates are identical (more than 99%). The identical percentage of Gag, Pol, Pro and Env at protein level between Shaanxi1 to 4 are all more than 98%. A consensus sequence for the Shaanxi ENTV-2 genome was determined determined (ENTV-2-Shaanxi1) and compared to that of ENTV-2 (accession no. AY197548.1), ENTV-2-SC (accession no. HM104174.1), ENTV-1-1NA1(accession no. GU292317.1), and JSRV-USA (accession no. NC-001494.1) using MegAlign program in DNASTAR. For full genome, ENTV-2-Shaanxi1 is 99.0% identical at the nucleotide level to ENTV-2-SC, and 89.6% identical at the nucleotide level to ENTV-2 (Table 4).

**Table 4:** Comparison of percentage nucleotide (Ntd) and amino acid (Aa) identity of the ENTV-2-Shaanxi1 consensus sequence with ENTV-2, ENTV-2-SC, ENTV-1-1NA1 and JSRV-USA

		ENTV-2	ENTV-2-SC	ENTV-1-1NA1	JSRV-USA
Full genome	Ntd	89.6%	97.0%	87.4%	87.5%
LTR	Ntd	99.0%	Not available	79.8%	76.0%
gag	Ntd	90.7%	99.4%	87.1%	86.6%
	Aa	96.6%	99.3%	91.8%	90.2%
pro	Ntd	90.0%	97.1%	92.3%	91.2%
	Aa	94.8%	97.7%	94.5%	93.8%
pol	Ntd	88.3%	98.4%	89.0%	92.1%
	Aa	92.3%	98.7%	94.5%	60.8%
env	Ntd	88.2%	99.8%	85.3%	83.7%
	Aa	94.9%	99.7%	88.1%	90.2%

### LTR

**Fig. 3:**
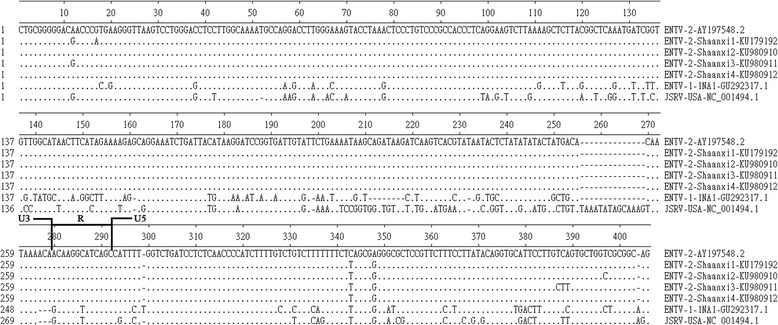
Alignment of the LTR nucleotide sequence of all four ENTV-2-Shaanxi isolates, ENTV-2, ENTV-1-1NA1, and JSRV-USA. Gaps in alignment are shown by dashes; dots represents identity. ENTV-2 was used as the reference sequence (top line)

The LTR of ENTV-2 Shaanxi isolates are 390 bases long. U3 is 265 bp, R is 13 bp, and U5 is 112 bp. The U3 region of the 5’LTR contains cisacting sequences necessary for viral replication and regulatory signals for retroviral transcription. The nucleotide differences of ENTV-2-Shaanxi1 ~ 4 to ENTV-2 (accession number AY197548.1) is 4, 3, 6, 2, respectively. Among them, two of the nucleotide differences common to all of the ENTV-2 Shaanxi isolates relative to ENTV-2 (accession number AY197548.1). There are more sequence differences in LTR between ENTV-2-Shaanxi and the other two virus (ENTV-1-1NA1, JSRV-USA), especially in the U3 (Fig. 3).

### Gag

**Fig. 4:**
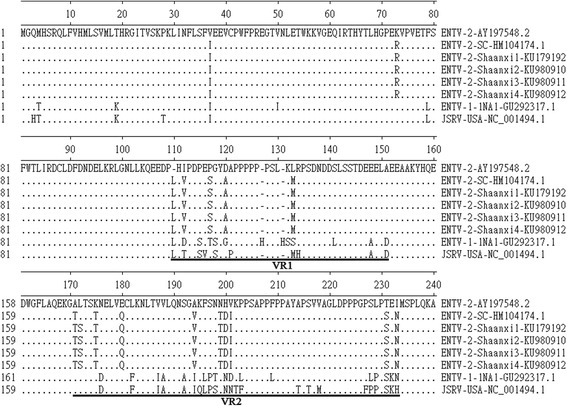
Gag alignment. DNAMAN alignment of the predicted amino acid sequence of the amino terminal portion of the Gag polyprotein from four ENTV-2-Shaanxi isolates, ENTV-2-SC, ENTV-2, ENTV-1-1NA1, and JSRV-USA. Gaps in alignment are shown by dashes; dots represents identity. ENTV-2 was used as the reference sequence (top line).VR1 and VR2 regions are underlined

The *gag* open reading frame of ENTV-2-Shaanxi1 ~ 4 encode a 612-amino-acid (aa) polypeptide (predicted molecular mass, 68 kDa). There are two sequences of Cys-X2-Cys-X4-His-X4-Cys (509 to 522 aa is CFVCGQPGHRAAVC, 536 to 549 aa is CPRCKKGKHWARDC) [8, 11]. This is typical of a zinc finger domain and is thought to mediate nucleocapsid binding to the genomic RNA [8, 11]. The ENTV-2-Shaanxi1 ~ 4 consensus sequence and ENTV-2-SC Gag were 99.3% identical at the amino acid level. The few differences are localized to two regions that have previously been described as variable regions 1 and 2 (VR1 and VR2) (Fig. 4) [2, 12, 13]. There were only 1 amino acid differences between the two variable regions of ENTV-2-Shaanxi1 ~ 4 and ENTV-2-SC, and there were 13 amino acid differences between the two variable regions of ENTV-2-Shaanxi1 ~ 4 and ENTV-2 (accession number AY197548.1), three of which were located in the VR1, ten were located in the VR2 (Fig. 4). A stretch of 6 consecutive proline residues(Aa 122 ~ 127) exists in all ENTV-2 [12, 14]. The ENTV-1NA1 contains a stretch of 5 consecutive proline residues at the proline-rich region. JSRV-USA contains a stretch of 7 consecutive proline residues at the region (Fig. 4).

### Pro

The *pro* open reading frame of ENTV-2 encodes a bifunctional protease/dUTPase of 308 amino acids (predicted molecular mass, 33 kDa) [1]. As with other retroviruses, Pro is expressed as a polyprotein with Gag by a mechanism involving ribosomal frameshifting-the exact site of which and relative efficiency have yet to be determined [7]. ENTV-2-Shaanxi1 Pro was greater than 97.7% identical at the amino acid level to ENTV-2-SC, and 94.8% identical at the amino acid level to ENTV-2 (accession number AY197548.1). Overall, the Pro region of ENTV-2-Shaanxi1 ~ 4 were highly homologous to other ENTV-2 and showed very little nucleotide or amino acid variability.

### Pol

The pol protein of ENTV-2 encodes a 869-amino-acid (aa) polypeptide (predicted molecular mass, 99 kDa) [1]. The Pol protein is synthesized as a polyprotein with Gag-Pro and is cleaved by Pro to produce reverse transcriptase (RT) and integrase (IN) after virus assembly. As with pro, the *pol* genes of ENTV-2-Shaanxi isolates were highly homologous to that of the ENTV-2-SC with 98.7% amino acid identity. Nearly every amino acid difference was the result of a synonymous substitution.

### Orf-x

**Fig. 5:**
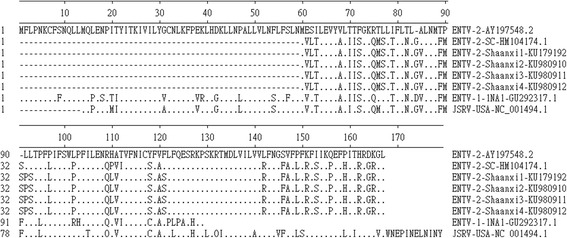
Orf-x alignment. Alignment of the predicted amino acid sequence of Orf-x from four ENTV-2-Shaanxi isolates, ENTV-2-SC, ENTV-2, ENTV-1-1NA1, and JSRV-USA. Gaps in alignment are shown by dashes; dots represents identity. ENTV-2 was used as the reference sequence (top line)

Although JSRV and ENTV-2 are classified as simple retroviruses, they both contain an additional open reading frame, termed Orf-x, entirely inside the *pol* gene [15]. For JSRV-USA(accession number NC_001494.1), the Orf-x encodes a 166 amino acids polypeptide [16]. ENTV-2 (accession number AY197548.1) Orf-x share the same initiating methionine as ENTV-1NA1 (accession number GU292317.1) but is truncated by a stop codon at amino acid 166. ENTV-2-Shaanxi1 ~ 4 Orf-x were truncated by a stop codons at amino acid 108 (Fig. 5). DNAMAN alignment of ENTV-2-Shaanxi1 ~ 4 Orf-x amino acid sequences revealed that ENTV-2-Shaanxi1 ~ 4 Orf-x initiates at the same methionine as ENTV-2-SC Orf-x and truncates at amino acid 108 (Fig. 5). The downstream sequences of ENTV-2-Shaanxi 1 ~ 4 Orf-x coincided with ENTV-2, ENTV-2-SC, ENTV-1-NA1 and JSRV-USA. Several amino acid differences were detected within Orf-x when individual ENTV-2-Shaanxi1 ~ 4 sequences were compared to each other. The amino acid sequence divergence among the ENTV-2-Shaanxi1 ~ 4 were much greater in Orf-x than in any other viral protein (Fig. 5).

### Env

**Fig. 6:**
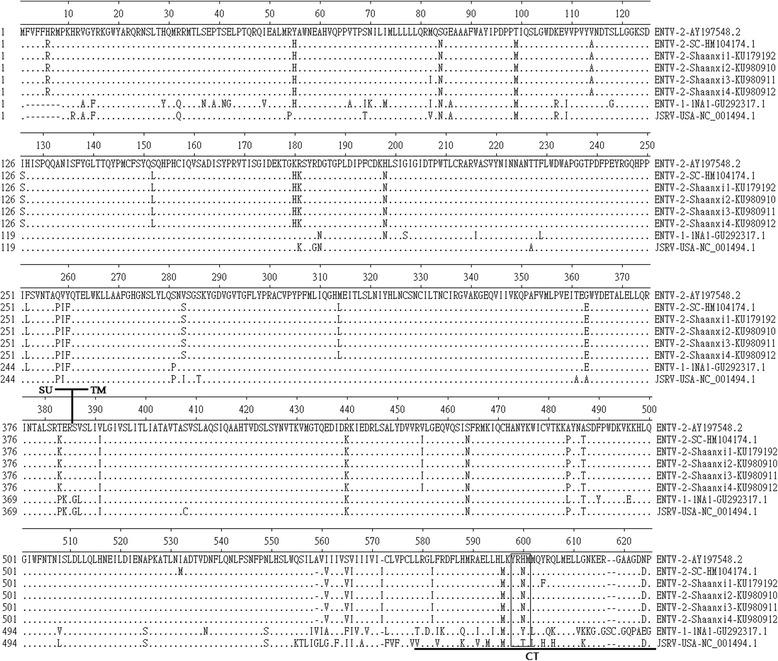
Envelope protein alignment. Alignment of the envelope protein amino acid sequence from four ENTV-2-Shaanxi isolates, ENTV-2-SC, ENTV-2, ENTV-1-1NA1, and JSRV-USA. Elbow bend arrows indicate the predicted cleavage sites between the SU and TM domains. The putative YXXM motif is outlined with boxes. Gaps in alignment are shown by dashes; dots represents identity. ENTV-2 was used as the reference sequence (top line)

ENTV *env* open reading frames encode a polypeptide precursor of 622 aa (predicted molecular mass, 71 kDa) which is cleaved twice, first following the peptide signal sequence and second following an internal proteolytic cleavage site, which generates the surface (SU, 1 ~ 385aa) and transmembrane (TM, 386 ~ 622aa) envelope proteins. There were only three amino acid differences between the Env proteins of ENTV-2-Shaanxi1 ~ 4 and ENTV-2-SC, one of them (82 Aa) were located in the signal peptide region(SP, 1 ~ 87aa), two of them were located in the transmembrane (TM) domain (Fig. 6). One amino acid differences was in the cytoplasmic tail (CT). There were 34 amino acid differences between the Env proteins of ENTV-2-Shaanxi1 ~ 4 and ENTV-2 (accession number AY197548.1), 4 of which were located in SP region, 17 of which were located in the surface (SU) domain. YXXM motif is a reliable molecular marker for the infectious exogenous virus. The cytoplasmic tail of the envelope transmembrane (TM) protein is necessary for transformation, and in particular a consensus binding motif (YXXM) for phosphatidylinositol 3-kinase (PI3K) is important [17]. The putative YXXM motif is outlined with boxes (Fig. 6). All the virus (ENTV-2-Shaanxi isolates, ENTV-2-SC, ENTV-2, ENTV-2-SC, ENTV-1-1NA1, and JSRV-USA) have the YXXM motif. The three tyrosine residues (Y590, Y592, and Y596), especially the Y590 in the ENTV-1-NA1 (accession number GU292317.1) CT are known to be essential for Env mediated transformation [18], but only two tyrosine residues (Y598 and Y602) were found among ENTV-2-Shaanxi2 ~ 4 isolates, and one tyrosine residues (Y598) were found in ENTV-2-Shaanxi (Fig. 6).

### Phylogenetic analysis of small ruminant betaretroviruses

**Fig. 7:**
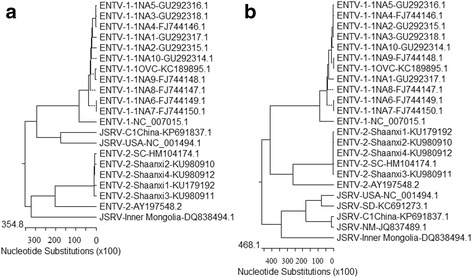
Phylogenetic analysis of caprine and ovine betaretroviruses. Phylogenetic analysis of the evolutionary relationship between the (**a**) *gag* gene sequence and the (**b**) *env* gene sequence of ENTV-2, ENTV-1 and JSRV isolates. Phylogenetic analyses were conducted using MegAlign and all positions containing gaps and missing data were eliminated from the dataset

Comparison of the nucleotide and amino acid sequence of the *gag* and *env* regions revealed that ENTV-2-Shaanxi1 is highly homologous to ENTV-2-Shaanxi3, and ENTV-2-Shaanxi2 is highly homologous to ENTV-2-Shaanxi4. For *gag* genes, the four ENTV from Shaanxi were greater than 99.3% identical at the nucleotide level and greater than 99.4% identical at the amino acid level to ENTV-2-SC. The four ENTV from Shaanxi were greater than 90.7% identical at the nucleotide level and greater than 96.6% identical at the amino acid level to ENTV-2 (accession number AY197548.1) (Fig. 7a). The *env* gene phylogenetic tree was similar to that of the *gag* gene. The sequences of the ENTV-2-Shaanxi1 ~ 4 were also in a large cluster. The ENTV-2-Shaanxi1 ~ 4 were greater than 99.8% identical at the nucleotide level and 99.7% identical at the amino acid level to ENTV-2-SC, the four ENTV from Shaanxi were greater than 88.2% identical at the nucleotide level and greater than 94.9% identical at the amino acid level to ENTV-2 (accession number AY197548.1) (Fig. 7b). Phylogenetic analysis showed ENTV-2-Shaanxi1 ~ 4, ENTV-2 (accession number AY197548.1) and ENTV-2-SC of China were in the same branch.

## Discussion

**Fig. 8:**
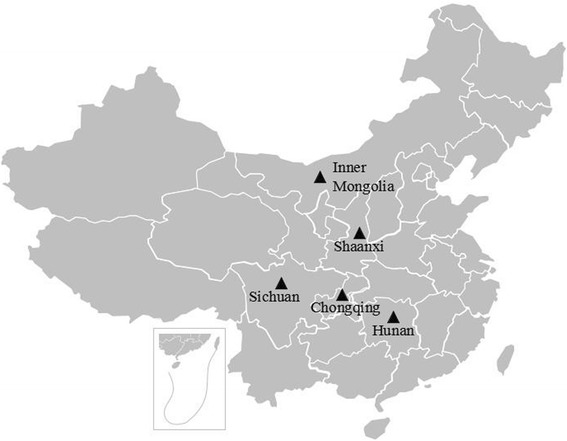
The endemic of ENA in China. Endemic areas were listed in the picture

Enzootic nasal adenocarcinoma occurs naturally in all continents except Australia and New Zealand [19]. The disease was popular in many goat breeding areas in China. From 1980s, the disease has been found in Inner Mongolia, Hunan, Chongqing, Sichuan in turn [20–23]. In 2015, the disease has been found in several areas in Shaanxi province which locates near endemic areas of China (Fig. 8). So far, there is no related reports in other areas. ENA outbreak areas distributed in the Northern or Midwest of China, where are the main breeding areas of goats. The incidence of ENTV infection in small ruminants of China requires further investigation.

These features of ENA in Shaanxi province are similar to bighorn sheep (*Ovis canadensis*) sinus tumors [24, 25]. Clinically, domestic sheep and goats affected by ENA have abundant seromucinous nasal discharge. Similarities between ENA and bighorn sheep sinus tumors include the presence of seromucinous nasal discharge clinically, the gross finding of a soft white mass in the sinus cavity, and classification of some masses as adenocarcinoma [24, 26]. Additionally, the inflammatory nasal polyps often associated with ENA share characteristics with the hyperplastic masses described here for bighorn sheep, although in bighorn sheep the sinus tumors is a diffuse thickening of the sinus lining and not a discrete polypoid mass. Prominent differences between ENA and the sinus tumors described here are location (sinus tumors in bighorn sheep and nasal in domestic sheep and goats) and malignancy (predominantly, hyperplastic masses in bighorn sheep and neoplastic masses in domestic sheep and goats). Other prominent differences between the 2 entities include the papillary appearance and often grey-red color of ENA tumors not characteristic of bighorn sheep sinus tumors, as well as the prominent mesenchymal population histologically present in bighorn sheep sinus tumors but infrequently described for ENA [24].

This study represented four full-length ENTV-2 sequences from goat flocks in Shaanxi province of China, and made genomic analysis with other betaretrovirus genus. Understanding the genetic heterogeneity of ENTV-2 is important both as a tool for epidemiological studies and as means to clarify the origin and future evolution of ENTV-2 [7]. The molecular sequence of this virus is closely related to sequences of JSRV and ENTV-1 [11, 27]. The ENTV-2-Shaanxi1 ~ 4 genome shared greater than 97.0% with ENTV-2-SC (accession number HM104174.1), and 89.6% sequence identity with the ENTV-2 sequence (accession number AY197548.1). ENTV-2-Shaanxi1 ~ 4 and ENTV-2-SC differ significantly to the ENTV-2 sequence (accession number AY197548.1). The isolation between different continents probably leads to the relatively large difference. ENTV-2 is closely related to ENTV-1 which is associated with enzootic adenocarcinoma of sheep, and to jaagsiekte retrovirus. The main sequence differences between these viruses resided in LTR, Orf-x, two small regions in Gag and the transmembrane (TM) region of Env. The LTR of ENTV-2-Shaanxi1 ~ 4 were similar to ENTV-2. The role of ENTV LTR in pathogenesis has yet to be determined. There was very little amino acid diversity in the Gag polyprotein except in the two domains defined as variable (VR1 and VR2) [12], suggesting that this part of the genomemay be able to withstand change. A stretch of 6 consecutive proline residues(Aa 122 ~ 127) exists in all ENTV-2. JSRV-USA contains a stretch of 7 consecutive proline residues at the region. To JSRV, the PPPPPPPS motif of the exogenous VR1 is neither necessary nor sufficient for particle release [12, 14]. Maybe the fuction of the proline-rich region in ENTV-2 is similar with JSRV. Orf-x is the most genetically diverse protein coding sequence of ENTV-2. Most of the amino acid differences were found in Orf-x, which in the corresponding ENTV-2-Shaanxi1 ~ 4 and ENTV-2-SC genome were 108 amino acids, but ENTV-2 sequence (accession number AY197548.1) is 166 amino acids. ENTV-2 in China potentially encode a truncated Orf-x protein compared to ENTV-2 (accession number AY197548.1). The same situation also appears in ENTV-1. Orf-x is the most genetically diverse protein coding sequence of ENTV-1 [7]. One study showed that a complete Orf-x is not required for pathogenesis of JSRV [28]. Truncation of Orf-x in JSRV did not alter the pathogenesis of the virus compared to wild type JSRV in experimental infection. So the large genetic changes of Orf-x in ENTV-2-Shaanxi may indicate that Orf-x does not play a significant role in in the pathogenesis of ENA. The sequence differences of Orf-x between different strains may be due to the regional differences. ENTV-2 is also more like JSRV than ENTV-1 in the C-terminal region of the Env TM cytoplasmic tail, so it is possible that some aspect of this region is also important in dissemination. Apart from Orf-x, two small regions in Gag, the transmembrane (TM) region, especially the cytoplasmic tail (CT) of Env, were the main differences between ENTV-2-Shaanxi1 ~ 4 and other ENTV-2. ENTV-2-Shaanxi1 ~ 4 were found to be highly conserved with less than 3.4% amino acid differences and less than 9.3% nucleotide differences in the *gag* coding region to ENTV-2 and ENTV-2-SC, and less than 0.3% amino acid differences and less than 5.1% nucleotide differences in the *env* coding region to ENTV-2-SC. The three tyrosine residues (Y590, Y592, and Y596), especially the Y590 in the ENTV-1NA1 (accession number GU292317.1) CT are known to be essential for Env mediated transformation [17, 18, 29], but only two tyrosine residues (Y598 and Y602) were found among ENTV-2-Shaanxi2 ~ 4 isolates, and one tyrosine residues (Y598) were found in ENTV-2-Shaanxi. Y590 residue is essential for tumorigenesis and/or replication of JSRV [29]. Maeda and others highlighted the importance of a single tyrosine residue in the cytoplasmic tail of JSRV TM, Y590. The Y590 is conserved in the ENTV-1 cytoplasmic tail, and mutation of this residue greatly reduces transformation; in contrast, mutation of two other tyrosine residues in the ENTV-1 TM (Y592 and Y596) had relatively less effect [17, 18, 29]. Similarly, the Y598 of ENTV-2 is corresponds to Y590 of ENTV-1 or JSRV. The Y598 is conserved in ENTV-2 cytoplasmic tail. Except ENTV-2-Shaanxi, the other five ENTV-2 sequences have another tyrosine residue Y602. Y590 residue is essential for tumorigenesis and/or replication of JSRV. It likely that only Y598 is important to signal transduction for transformation of ENTV-2. The cytoplasmic tail of the envelope transmembrane (TM) protein is necessary for transformation, and in particular a consensus binding motif (YXXM) for phosphatidylinositol 3-kinase (PI3K) is important. YXXM motif is a reliable molecular marker for the infectious exogenous virus [18]. All the ENTV-2-Shaanxi isolates have the YXXM motif. Maybe the YXXM motif is an essential part for transformation, rather than single amino acids.

The importance of dissemination in the virus life-cycle/pathogenesis remains to be proven, particularly as ENTV-1 and ENTV-2 induce very similar diseases even though dissemination in the host appears to be more limited for ENTV-1 [1]. Future efforts will involve application of our RT-PCR protocol for preclinical diagnosis of ENTV-2 from nasal swabs and construction of an infectious molecular clone of ENTV-2 for subsequent pathogenesis studies. The next studies of molecular epidemiological characterization of ENTV-2 isolates will increase our understanding and will provide knowledge on how to achieve a better understanding of pathways that have led to later transmission of ENTV-2 and how control management should be implemented to prevent the spread of disease.

## Conclusions

In this study, the complete sequences were determined from four isolates of Shaanxi province. The ENTV-2-Shaanxi genomes shared 97.0% sequence identity with ENTV-2-SC (accession number HM104174.1), and 89.6% sequence identity with the ENTV-2 sequence (accession number AY197548.1). ENTV-2 is closely related to the ENTV-1 and JSRV. The main sequence differences between these viruses reside in LTR, VR1 and VR2 of Gag, Orf-x, and the transmembrane (TM) region of Env. Phylogenetic analysis by nucleotide sequences showed that four ENTV-2 isolates of shaanxi province were closest related to three ENTV-2 isolates published in NCBI, especially with ENTV-2-SC strain. This finding indicates that ENA most likely was introduced to Shaanxi province by the movement of contaminated goats from other areas in China. This study adds to understand the circulation, variation and distribution of ENTV-2, and may prove beneficial in future control or eradication programmes.
